# Paraneoplastic Pemphigus: A Striking Complication of Undiagnosed Lymphoma

**DOI:** 10.1155/2022/3641474

**Published:** 2022-10-25

**Authors:** Blake O. Adnani, Kathleen O' Brien, Zaw W. Myint, Brandon L. Adler

**Affiliations:** ^1^Department of Internal Medicine, Keck School of Medicine, University of Southern California, Los Angeles, USA; ^2^Department of Dermatology, Keck School of Medicine, University of Southern California, Los Angeles, USA; ^3^Department of Hematology, Keck School of Medicine, University of Southern California, Los Angeles, USA

## Abstract

A 49-year-old male with no past medical history presented with acute-onset painful mucosal erosions along with flaccid bullae on his trunk, scalp, and intertriginous areas. The patient initially underwent a skin biopsy which demonstrated suprabasilar acantholysis and lichenoid interface dermatitis. This was followed by a computed tomography scan which identified a large abdominal lymph node. Core needle biopsy of this node demonstrated follicular lymphoma. Lastly, indirect immunofluorescence (IIF) in rat bladder was positive (titer 1 : 10,240). This finding confirmed the diagnosis of paraneoplastic pemphigus (PNP) in the setting of follicular lymphoma. The patient's cutaneous disease was treated with a combination of intravenous immunoglobulin and methylprednisolone, along with intravenous rituximab, with a resolution of his cutaneous symptoms. His lymphoma was treated with six cycles of rituximab, cyclophosphamide, doxorubicin, vincristine, and prednisone (R–CHOP), with an interval decrease in his tumor burden. PNP is an autoimmune-mediated mucocutaneous disease associated with underlying neoplasm, most commonly non-Hodgkin lymphoma or chronic lymphocytic leukemia. Affected patients develop variable autoantibodies to antigens on keratinocytes and the basement membrane zone. Severe intractable stomatitis is characteristic, in addition to polymorphous cutaneous eruptions including bullae and erosions. Mortality rates can reach up to 90% due to malignancy, sepsis, or bronchiolitis obliterans, an irreversible and often lethal cause of pulmonary insufficiency. We highlight PNP manifesting in a patient with lymphoma, who responded well to the skin- and malignancy-directed treatments. PNP is an exceedingly rare diagnosis that should be considered in a patient with intractable stomatitis.

## 1. Case Presentation

**Figure 1:**
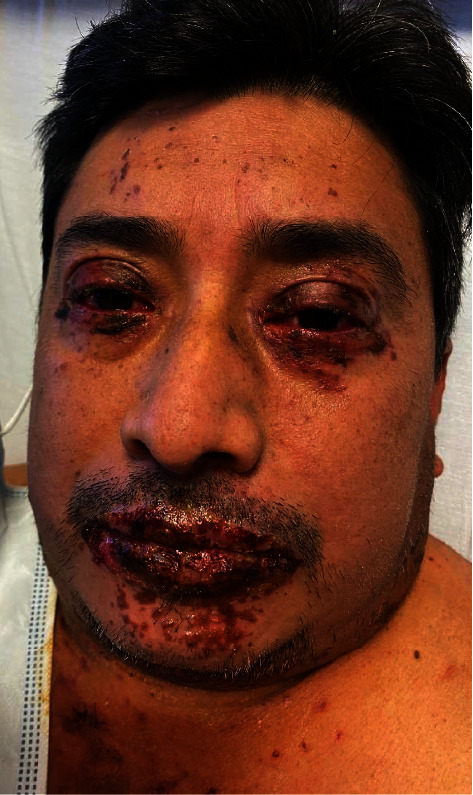
Mucocutaneous involvement in the initial presentation.

**Figure 2:**
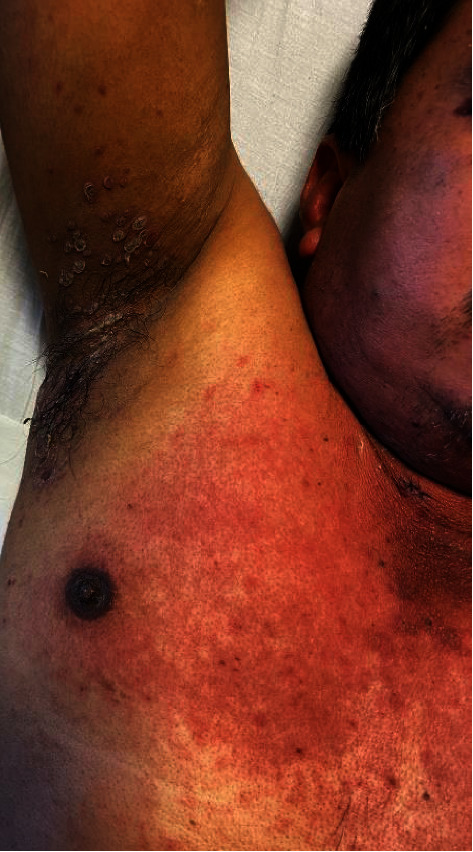
Axillary involvement in the initial presentation.

A 49-year-old male presented with acute-onset painful oral, genital, and ocular erosions along with dusky erythema and flaccid bullae on his trunk, scalp, and intertriginous areas (Figures 1 and 2). Ten days prior to presentation, the patient noted bilateral eye pain and redness. Two days after these initial symptoms, he developed dysphagia and painful oral lesions, which made it difficult for him to ingest food and water. The following day, he developed tender, painful perianal lesions. He denied any past medical history, recent travel, medication or illicit drug use, or infectious symptoms. Physical exam was notable for prominent bilateral conjunctival injection, bilateral periorbital erythema and ulcerations, perioral hemorrhagic crusting, tender perianal ulcerations, and a diffuse macular rash on the trunk along with multiple nontender papules in the bilateral axilla. Initial labs (including complete blood count, comprehensive metabolic panel, and peripheral blood cultures) were unremarkable.

**Figure 3:**
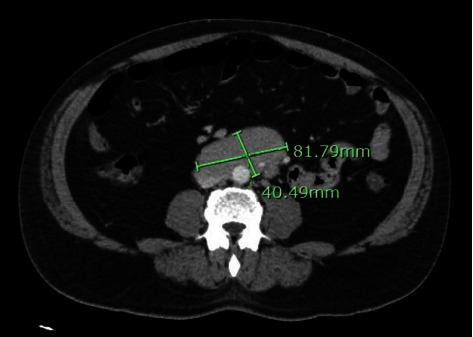
CT Abdomen and Pelvis with contrast showing enlarged preaortic lymph node (measured as 8 × 4 cm on axial view).

**Figure 4:**
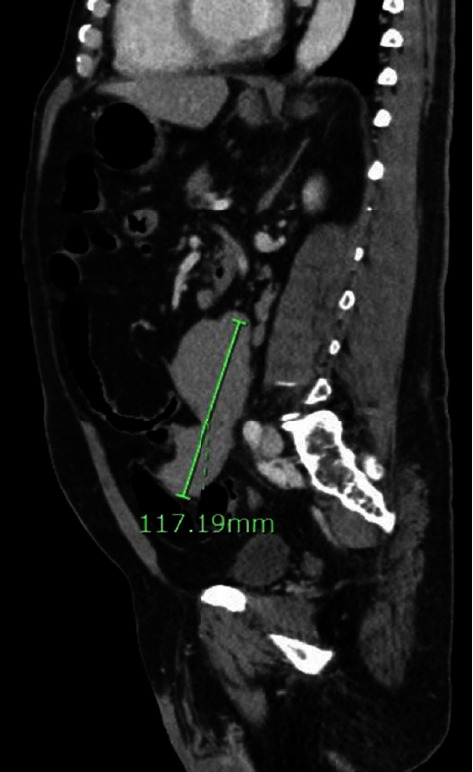
The same mass that was visualized in Figure 3, measured as 11.7 cm on sagittal view of CT.

Dermatology was consulted, and a skin biopsy demonstrated suprabasilar acantholysis with lichenoid interface dermatitis. Serologic studies, including enzyme-linked immunosorbent assays (ELISA), showed autoantibodies to bullous pemphigoid antigen 1 along with desmogleins 1 and 3. Computerized tomography (CT) demonstrated an eleven-centimeter preaortic lymph node in the abdomen (Figures 3 and 4), along with scattered retroperitoneal and mesenteric lymphadenopathy, Positron emission tomography (PET-CT) confirmed increased fluorodeoxyglucose (FDG) uptake in these areas. Core needle biopsy of the enlarged preaortic node revealed follicular lymphoma (CD10+, CD20+, BCL2+, and BCL6+ by immunohistochemistry). A paraneoplastic pemphigus panel displayed positive indirect immunofluorescence (IIF) on rat bladder (titer 1 : 10,240), mouse bladder (titer 1: 2,560), mouse heart (intercalating disks, titer 1 : 80), and monkey esophagus (cell surface). These findings confirmed the diagnosis of paraneoplastic pemphigus (PNP).

**Figure 5:**
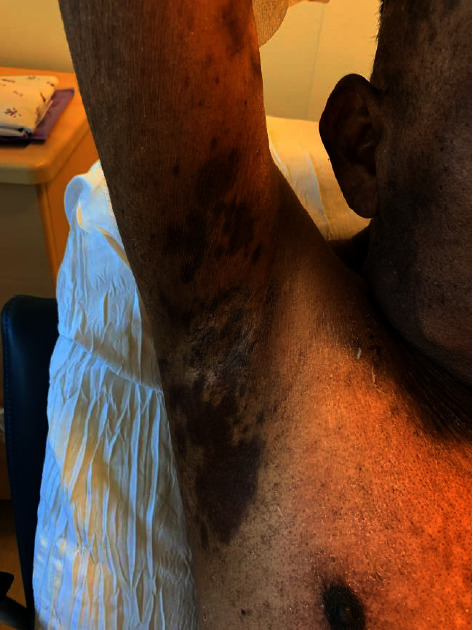
Resolution of axillary involvement after two months of treatment.

**Figure 6:**
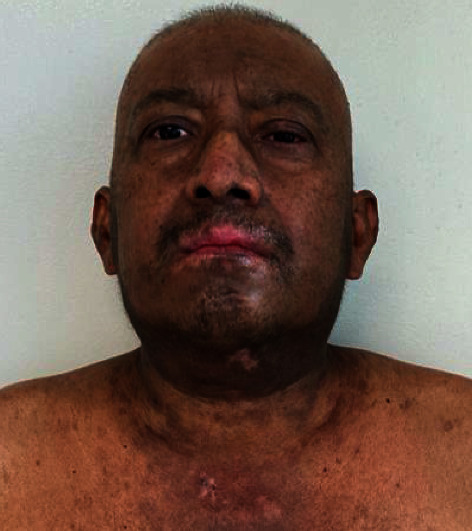
Resolution of oral and mucocutaneous involvement after four months of treatment.

The patient's cutaneous disease was treated with 1 mg/kg/day of intravenous immunoglobulin for three days, 2 mg/kg/day of intravenous methylprednisolone for two weeks (followed by a two-month oral prednisone taper), and four doses of weekly 375 mg/m2 intravenous rituximab. He had a resolution of his skin lesions after two months of treatment (Figure 5), but his oral lesions persisted. His lymphoma was treated with six cycles of rituximab, cyclophosphamide, doxorubicin, vincristine, and prednisone (R-CHOP), with an interval decrease in his tumor burden. His oral lesions ultimately resolved about four months after the initiation of treatment (Figure 6).

## 2. Discussion

PNP is an autoimmune-mediated mucocutaneous disease associated with underlying neoplasm, most commonly non-Hodgkin lymphoma or chronic lymphocytic leukemia. Affected patients develop variable autoantibodies to antigens on keratinocytes and the basement membrane zone [1]. Severe intractable stomatitis is characteristic, in addition to polymorphous cutaneous eruptions including bullae and erosions. Approximately one-third of patients with PNP present with new mucocutaneous lesions prior to diagnosis of their underlying neoplasia [1].

IIF using rat bladder is specific for PNP and differentiates it from pemphigus vulgaris [2]. However, previous studies have shown that IIF can be positive in roughly 20% of patients with pemphigus vulgaris and pemphigus foliaceus [3]. Mortality rates can reach up to 90% due to malignancy, sepsis, or bronchiolitis obliterans, an irreversible and often lethal cause of pulmonary insufficiency [4–6].

PNP should not be confused with other paraneoplastic dermatoses, such as paraneoplastic dermatomyositis (PND) or necrolytic migratory erythema (NME). NME is characterized by peripheral vesicles and crusts, with central pigmentation in an annular pattern. It is often associated with glucagonomas and inflammatory bowel disease [7]. In contrast, PND presents as a heliotrope rash with splinter hemorrhages and is often associated with lung, breast, and ovarian cancer. The Sweet syndrome presents similarly to PND, with tender, erythematous papules and nodules. However, Sweet syndrome can be differentiated from PND through biopsy demonstrating a large proportion of mature neutrophils infiltrating the dermis [8, 9].

We highlight PNP manifesting in a patient with follicular lymphoma, who responded well to the skin- and malignancy-directed treatments. PNP is an exceedingly rare diagnosis that should be considered in a patient with intractable stomatitis.

### 2.1. Clinical Question

A 44-year-old male presents with sudden-onset intractable mucositis and flaccid bullae on his trunk. He is ultimately diagnosed with lymphoma and initiated on the appropriate treatment, but later develops acute hypoxic respiratory failure of unclear origin. What is the most likely diagnosis?

  Stevens–Johnson syndrome (SJS)
  Paraneoplastic pemphigus (PNP)
  Pemphigus vulgaris
  Sweet syndrome
  Necrolytic migratory erythema (NME)

### 2.2. Answer

The correct answer, in this case, is paraneoplastic pemphigus. Bronchiolitis obliterans is an irreversible and often lethal cause of pulmonary insufficiency in patients who have been diagnosed with paraneoplastic pemphigus. The exact pathophysiology is unclear, but it is presumed that some of the autoantibodies generated by PNP play a role in causing acute inflammation of the respiratory epithelium [8]. Clinicians taking care of patients diagnosed with paraneoplastic pemphigus should have a high degree of suspicion for bronchiolitis obliterans in the case of sudden respiratory decline.

While both NME and Sweet syndrome are paraneoplastic dermatoses associated with underlying malignancies, neither is associated with significant pulmonary pathology. SJS can also cause lung disease leading to respiratory failure, but the patient's history of malignancy in the setting of skin erosions should lead you to consider the diagnosis of paraneoplastic pemphigus.

## Data Availability

No underlying data was collected or produced in this report.
